# Single-cell analysis of T lymphocytes infiltrating colorectal carcinoma: the dilemma of specificity

**DOI:** 10.1080/2162402X.2023.2300520

**Published:** 2024-01-04

**Authors:** Manuela Lizarralde-Guerrero, Laura Zucaro, Guido Kroemer, Jonathan G. Pol

**Affiliations:** a Centre de Recherche des Cordeliers, Equipe labellisée par la Ligue contre le cancer, Université de Paris Cité, Sorbonne Université, Inserm U1138, Institut Universitaire de France, Paris, France; b Metabolomics and Cell Biology Platforms, Institut Gustave Roussy, Villejuif, France; c Université Paris-Saclay, Faculté de Médecine, Kremlin-Bicêtre, France; d Department of Translational Medical Sciences, University of Campania ‘Luigi Vanvitelli’, Naples, Italy; e Institut du Cancer Paris CARPEM, Department of Biology, Hôpital Européen Georges Pompidou, AP-HP, Paris, France

**Keywords:** Colorectal cancer, microsatellite instability, single-cell transcriptomics, T cell receptor, tumor-infiltrating lymphocytes

## Abstract

Advances in single-cell RNA and T cell receptor (TCR) sequencing allow to study the specificity and functionality of tumor-infiltrating T lymphocytes. A recent study unravels fundamental differences between microsatellite-instable (MSI) colorectal cancers, in which T cells tend to be tumor-specific, and microsatellite-stable (MSS) cancers, in which T cells exhibit bystander features.

## Main text

Colorectal cancer (CRC) is a heterogeneous disease with a variable degree of aggressiveness and sensitivity to treatments that is dictated by tumor cell intrinsic and extrinsic features.^1^ These include genetic traits of malignant cells, their surrounding immune microenvironment or the host gut microbiota. In-depth characterization of these factors has bearing for the clinical management of CRC, from diagnosis and prognosis to therapeutic decision making.

In addition to the tumor/node/metastasis (TNM) staging system, therapeutic guidance of CRC in Western countries relies on the detection of DNA mismatch repair deficiency (MMRd) and the resulting microsatellite instability (MSI). On one side, MMRd is diagnosed by immunohistochemistry in tumor biopsies. Failure to detect some proteins involved in MMR (e.g., MLH1, MSH2) indicates the MMRd/MSI subtype. On the other side, the MSI phenotype is established after detection by PCR of variations in the length of microsatellite-enriched regions. As opposed to CRC with MMR proficiency (MMRp) and microsatellite stability (MSS), colorectal malignancies with MMRd/MSI status harbor a higher tumor mutational burden (TMB). This particularity increases cancer antigenicity and hence influences tumor surveillance by adaptive immune effector cells as well as the response to immune checkpoint inhibitors (ICIs).^2,3^ Along this line, the Immunoscore (IS) has been internationally validated for refining the prognosis of CRC. It consists in measuring the density of CD3^+^ T lymphocytes, and their cytotoxic CD8^+^ T cell subset, in both the center and invasive margin of the tumor. Patients with tumors characterized by a high IS, indicative of an immune-infiltrated or “hot” CRC, show extended survival and superior sensitivity to treatments, as compared to immune-desert/“cold” malignant tissues.^4,5^

Nonetheless, despite a lower neoantigen repertoire and a supposedly limited T cell infiltrate, some MSS CRC happen to respond to anti-PDCD1 (best known as PD-1) and anti-CTLA4 dual immunotherapy. Furthermore, the molecular hallmarks of CRC remain insufficient to differentiate durable responders from non-responders to PD-1 blockade in MSI CRC. These observations reveal a void in our current understanding of the clinico-immunological heterogeneity underlying MSI and MSS CRC.^6^

With the spreading adoption of next-generation sequencing and multi-omics workflows, together with artificial intelligence-assisted analyses, additional features of CRC are being identified, leading to improved patient stratification.^7–9^

As an illustration, microbial profiling using 16S ribosomal RNA (rRNA) sequencing unraveled an additional layer of immune regulation mediated by the gut microbiota. Several underlying mechanisms have been unveiled, including the supply of adjuvant compounds (e.g., microbe-derived lipopolysaccharides or metabolites) and the stimulation of cross-reactive effector T cells specific to viral, fungal, or bacterial epitopes shared with tumor antigens.^9^

In a recent study, Borràs DM and colleagues applied single-cell RNA and T cell receptor (TCR) dual sequencing (scRNA/TCR-seq)^10^ to delve into the intricate workings of CD8^+^ T cells in the context of CRC.^6^ Their findings unveiled a remarkable dichotomy in the behavior of CD8^+^ T cells, revealing distinct activation patterns and functional characteristics in MSI and MSS CRC subtypes.

**Figure 1.**
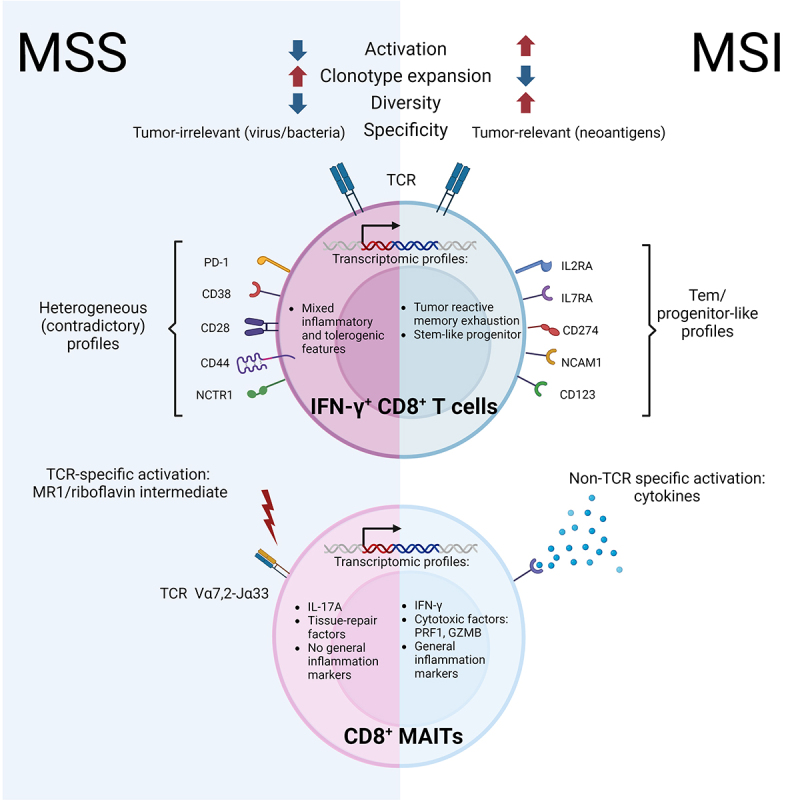
Divergent CD8^+^ T cell profiles explains differential cancer immunosurveillance potency in MSI and MSS CRC. In MSI, IFN-γ^+^ CD8^+^ T cells demonstrate strong activation, characterized by a limited but diverse expansion of TCR clonotypes specific to tumor antigens, compatible with efficient antitumor activity. This CD8^+^ T cell compartment includes MAIT cells expressing pro-inflammatory and cytotoxic factors (e.g., PRF1, GZMB). By contrast, IFN-γ^+^ CD8^+^ T cells enriched in MSS CRC show weaker and bystander activation, characterized by a potent expansion of a restricted number of TCR clonotypes mostly relevant to microbes, responsible for a “pseudo-hot” immune microenvironment that translates in ineffective antitumor immunity. Along this line, CD8^+^ MAIT cells populating MSS CRC express tissue-repair factors and IL-17A, reflective of an activation by bacterial elements (i.e., riboflavin-derived compounds presented onto MR1 non-classical MHC-I molecules). The transcriptomic signature of these activated CD8^+^ T cells is indicative of memory exhaustion and stem-like progenitor phenotypes in MSI tumors, as opposed to a heterogenous pro-inflammatory and tolerogenic phenotype in MSS CRC. High-plex mass cytometry comforted these findings at the protein level. On one side, immune profile heterogeneity was observed in MSS with detection of both co-inhibitory (e.g., PD-1) and stimulatory (e.g., CD28) immune markers on CD8^+^ T lymphocytes. On the other side, MSI-infiltrating CD8^+^ T cells display surface factors associated with an effector memory/progenitor-like profile (e.g., IL2RA, IL7RA, CD123, CD274). Created with Biorender.com. GZMB, granzyme B; IFN, interferon; IL, interleukin; MAIT, mucosal-associated invariant T cell; MHC, major histocompatibility complex; MR1, major histocompatibility complex, class I-related; MSI, microsatellite instability; MSS, microsatellite stability; NCAM1, neural cell adhesion molecule 1; NCTR1, natural cytotoxicity triggering receptor 1; PD-1, programmed death-1; PRF1, perforin 1; TCR, T cell receptor.

In MSI CRC, CD8^+^ T cells emerge as tumor-specific warriors, primed to recognize and eradicate cancerous cells (Figure 1). Their activation was characterized by a robust interferon-γ (IFN-γ) signaling response, indicative of effective antitumor immunity. This heightened signaling cascade fuels the expression of genes crucial for T cell cytotoxicity, enabling them to directly eliminate tumor cells. The arsenal of MSI CRC CD8^+^ T cells was further enhanced by a diverse TCR repertoire, reactive to a wide array of cancer (neo)antigens.^6^ This diversity ensures that even if certain tumor antigens are lost or mutated, the immune system retains the capacity to detect and eliminate the malignancy.^6^

In contrast to their MSI CRC counterparts, MSS CRC CD8^+^ T cells adopted a bystander-like role, exhibiting tumor-unspecific or irrelevant activation (Figure 1). Their IFN-γ signaling response was less pronounced, and their TCR repertoire appeared less diverse and largely virus-specific, limiting their ability to specifically target tumor cells.^6^ This bystander activation pattern is reminiscent of “pseudo-T cell hot” tumors, where T cell infiltration does not correlate with antitumor immune activity.^6^

Considering the rarity of public single-cell transcriptomics datasets, the signaling modules associated with tumor reactivity in the single-CD8^+^ T cell approach was used to design a bulk tumor transcriptome classification for CRC patients. This “Immune Subtype Classification” (ISC) successfully stratified CRC patients of the TCGA database into seven subtypes: two MSI (ISC1a with a stem-like progenitor/high inflammatory/pro-immunogenic phenotype, ISC1b with a signature reminiscent of wound healing) and five MSS (ISC2a, ISC2b and ISC2c with high, mid and low inflammatory signatures, respectively; ISC3a and ISC3b immune silent with a wound healing mid and high profile, respectively). Moreover, the ISC system showed promise as a machine learning-based score to differentiate ICI responders from non-responders in MSI CRC (Figure 1).^6^ However, comprehensive validation in larger cohorts with immunotherapy interventions will be necessary.

The divergent behavior of CD8^+^ T cells in MSI and MSS CRC underscores the need for personalized immunotherapy approaches that capitalize on the unique immune landscapes of each CRC subtype. For MSI CRC patients, immunotherapy strategies that augment the number and activity of tumor-specific CD8^+^ T cells hold promise. This could involve adoptive cell therapy, where engineered T cells are infused into patients to directly target tumor cells. Additionally, combination immunotherapy approaches, such as combining immune checkpoint blockade with other immune modulators, could further enhance the antitumor response in MSI CRC. In MSS CRC, immunotherapy strategies that foster tumor-specific CD8^+^ T cell responses may be more effective. This could involve manipulating the gut microbiome or targeting specific genes and proteins that regulate T cell activation and function.

Altogether, the study by Borràs DM et al. provides a compelling roadmap for understanding the diverse immune landscapes of CRC and paves the way for the development of personalized immunotherapy strategies. Careful adaptation of treatments to the unique CD8^+^ T cell dynamics of MSI and MSS CRC holds major promise.
